# Traumatic Complete Isolated Transection of a Female Urethra: A Report of a Rare Case

**DOI:** 10.7759/cureus.100064

**Published:** 2025-12-25

**Authors:** Simranpreet Singh Kahlon, Meet Sanghani, Sunil Gokhroo

**Affiliations:** 1 Urology, RNT (Rabindranath Tagore) Medical College, Udaipur, IND

**Keywords:** isolated urethral injury, pelvic fracture, urethral injury, urethroplasty, vaginal laceration

## Abstract

Urethral transection without associated pelvic bone fracture is an uncommon entity in females. There are no standard guidelines available informing about the management of such injuries, due to the limited number of cases.

We present a case of a 22-year-old female who sustained an isolated urethral injury after falling from a height and was successfully managed with prompt surgical repair. Urethral transection occurred along with anterior vaginal wall laceration, due to compression of tissues between the pubic bone and the hard object on the ground on which the impact occurred upon falling from a height. End-to-end urethroplasty was performed, and the patient was voiding normally at six-month follow-up.

## Introduction

Urethral injuries are uncommon in females, unlike in males. Even more rare is the occurrence of urethral injury in the absence of pelvic fracture [1]. Common causes include trauma and obstetric surgeries. Apart from being overlooked, such isolated injuries have been inadequately reported in the literature, and their management is poorly outlined.

## Case presentation

A 22-year-old lady presented to the Emergency Department six hours after falling from a tree of approximately 10 feet in height, sustaining injury to the vaginal introitus. She had profuse vaginal bleeding and difficulty in passing urine. She was hemodynamically stable on presentation, with no major injury to the head, chest, abdomen, or limbs. The pelvic compression test was negative, and X-rays of the pelvis ruled out a fracture.

Foley’s catheter was attempted in the emergency ward but failed, and urethral injury was diagnosed, associated with an anterior vaginal wall laceration. A decision was taken to repair the injury in the emergency operating room. There was a crescentic tear in the anterior vaginal wall, with complete transection of the distal urethra. The distal transected end was 1 cm proximal to the urethral meatus, and the proximal end was also identified by gently inserting a Foley’s catheter into it.

**Figure 1:**
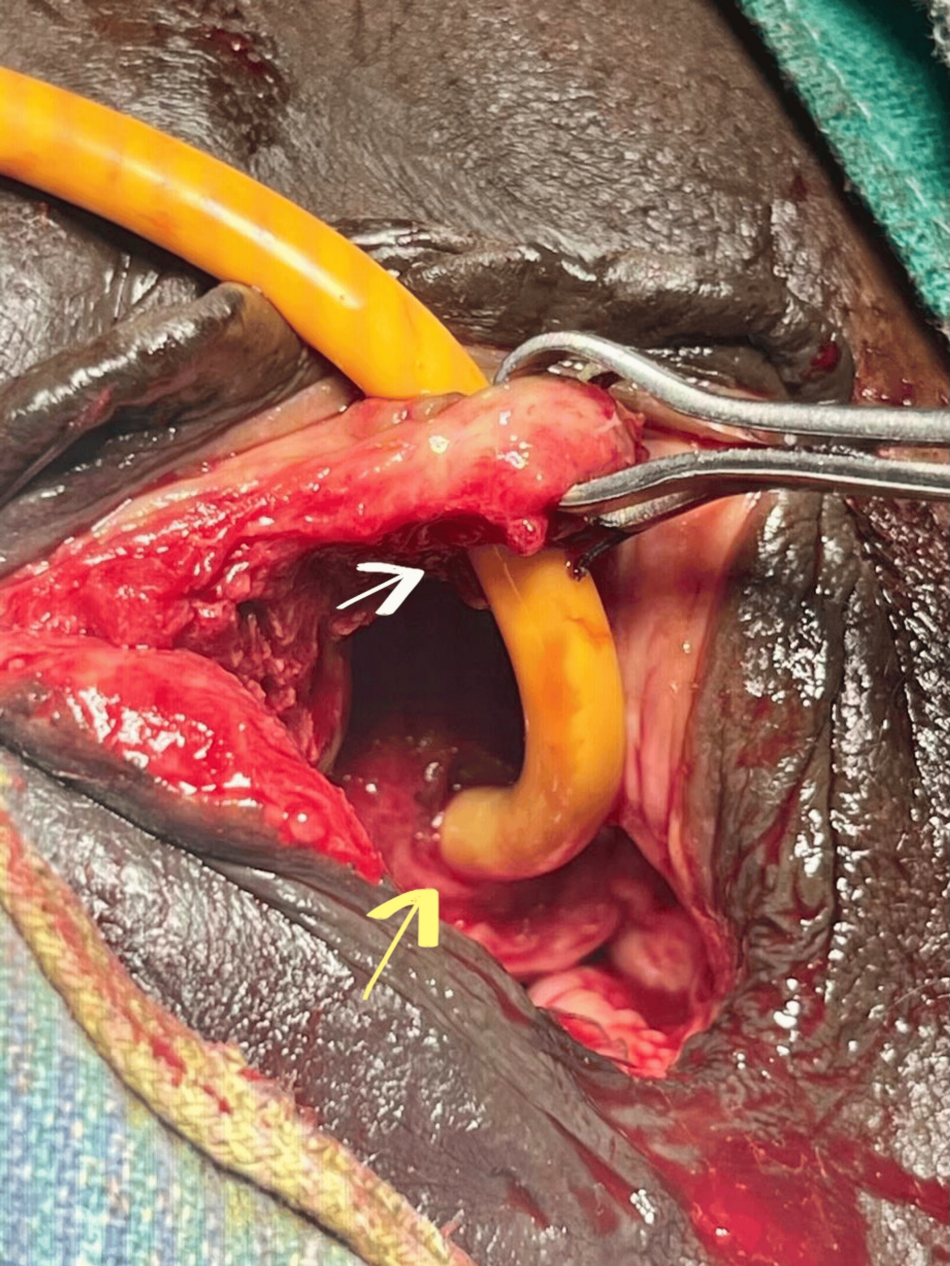
Severe laceration of anterior vaginal wall with complete avulsion of urethra Distal transected end of the urethra (white arrow) and proximal end of the urethra (yellow arrow).

**Figure 2:**
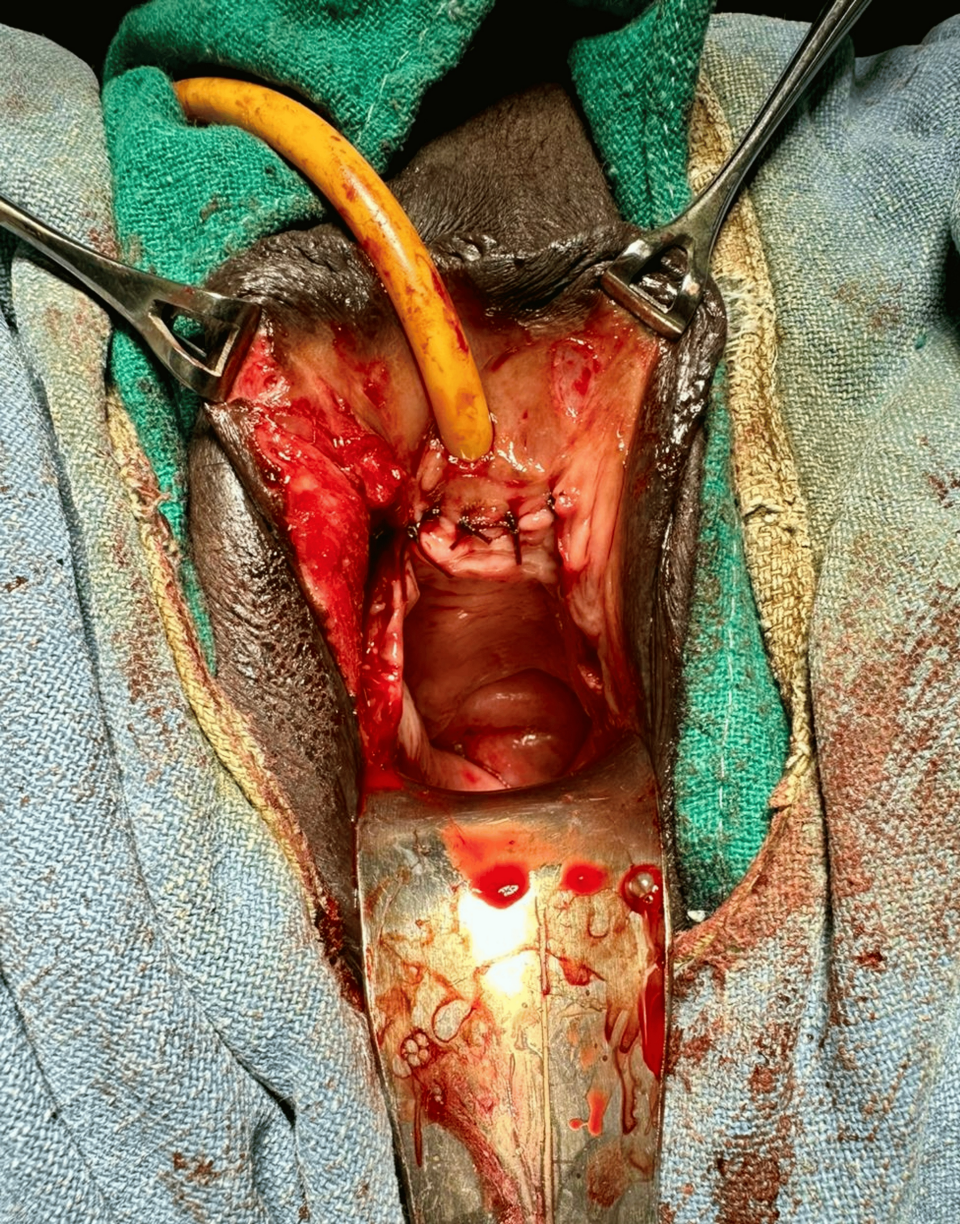
Wound after final closure

Urethral alignment was done by inserting a 16 Fr Foley’s catheter into the urinary bladder, and clear urine output was observed (Figure 1). Urethral margins were freshened, and end-to-end urethroplasty was done in a single layer with absorbable sutures over a 16 Fr catheter. Anterior vaginal wall repair was done with intermittent absorbable sutures (Figure 2).

**Figure 3:**
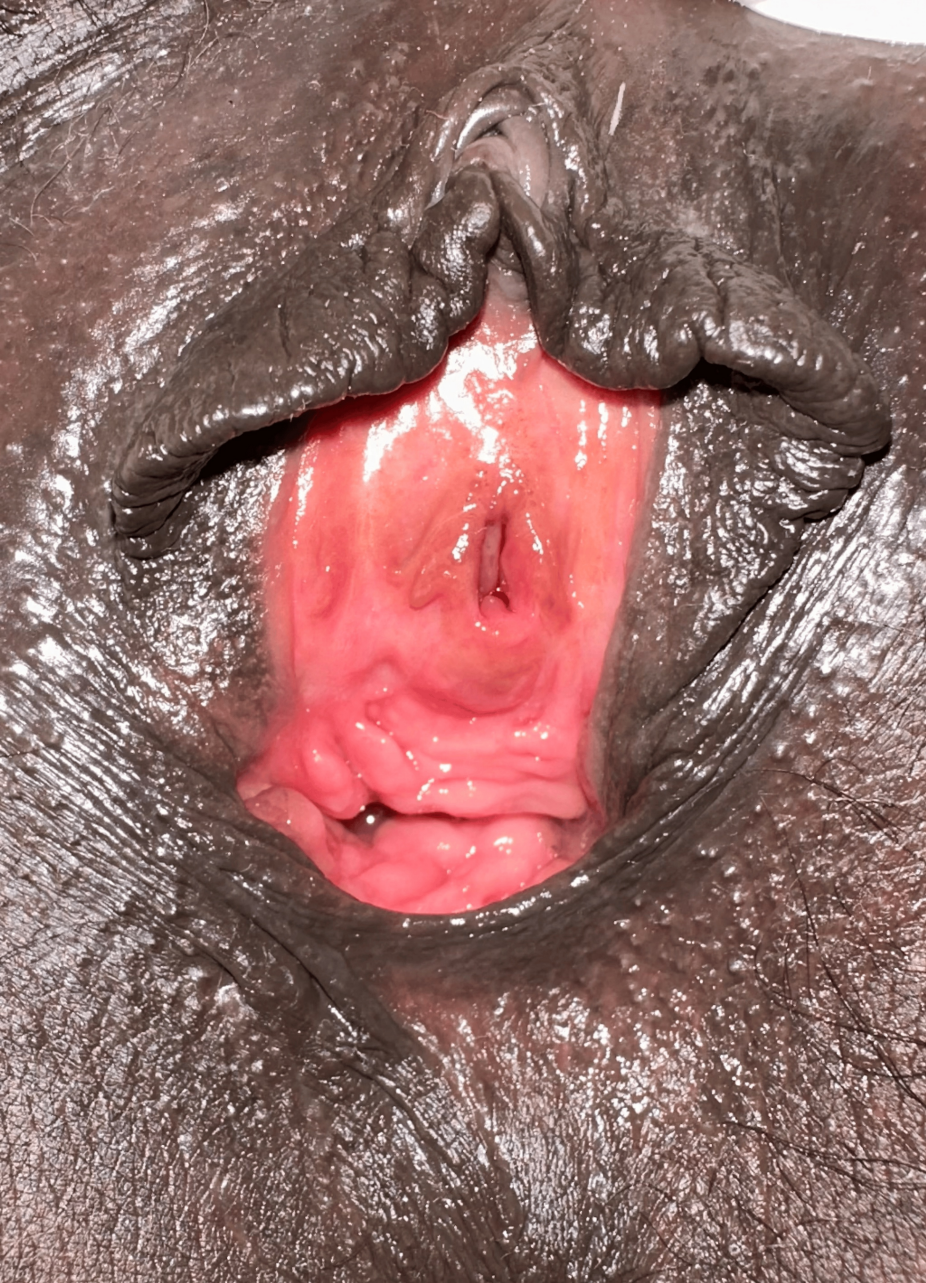
Completely healed wound six months after urethral repair with a healthy vagina

Foley’s urethral catheter was removed after two weeks, and the patient voided comfortably with a good stream. She had no difficulty in voiding, and there was no incontinence, with good wound healing at six-month follow-up (Figure 3).

## Discussion

Urethral injuries occur in approximately 10% of pelvic fractures in males, whereas in females, the incidence is lower, ranging from 4% to 6% [2]. Because of the shorter length of the female urethra and its course behind the pubic bone, it is protected from injuries in most cases. Isolated urethral injuries in females either require instrumentation during procedures involving the transvaginal route, or an impact causing compression of the urethra against the pubic bone, causing laceration of the anterior vaginal wall along with the urethra. Bull horn injuries are common in rural parts of India, and such injuries involving the urethra have also been reported in the literature [3]. The most pertinent question for the surgeon is whether to perform early repair or delayed repair of such injuries. Most of the case reports we reviewed favoured early repair, involving end-to-end anastomosis of transected urethral margins [2-4]. Another option is cystostomy, followed by delayed repair [4].

In our experience, primary repair can be safely carried out if done early following the injury. Freshening of urethral margins is also important for better results, as a lacerated wound is associated with contused tissue margins. Informed consent should be taken before the surgical repair because of the risk of incontinence, stricture, or urethrovaginal fistula formation.

## Conclusions

A very rare condition, isolated urethral trauma in women, is commonly overlooked in immediate post-injury evaluation. The optimal treatment for a complete urethral transection in this context involves swift diagnosis and immediate transvaginal repair to prevent subsequent morbidity. Follow-up should include addressing lower urinary tract symptoms and sexual function.
